# Novel Biologically Active Molecules, Biomaterials, and Nanoparticles for Microbial Biofilm Control in Human Medicine

**DOI:** 10.3390/molecules26092749

**Published:** 2021-05-07

**Authors:** Rossella Grande, Simone Carradori

**Affiliations:** Department of Pharmacy, University “G. d’Annunzio”, Chieti-Pescara, 66100 Chieti, Italy

The aim of the present special issue, proposed by two Co-Guest Editors with expertise in Clinical Microbiology and Medicinal Chemistry, is to collect and disseminate some of the most significant and innovative contributions focused on biofilm removal strategies, based on the use of natural or synthetic compounds/molecules/peptides or nanoparticles as well as biofilm formation inhibition aimed at both the control and monitoring of biofilm infections in medicine, food, industry, and natural environments. The increasing interest in the biofilm research field has been confirmed by the numerous articles in literature published in peer reviewed high-impact-factor journals such as Molecules. The peer review process allowed us to select 13 manuscripts out of 19 submitted by scientists from different countries that fit the scope of the research topic. We are grateful to all colleagues, Authors, and Reviewers that contributed to the realization of this special issue. In the same way, we thank MDPI and the Editorial Staff for their efficiency as well as for their constant and professional support.

Microbial biofilms are complex structures formed by cells embedded in an extracellular polymeric substance (EPS) matrix, a mixture of macromolecules such as exopolysaccharides; proteins; extracellular DNA; and, in some cases, outer membrane vesicles [1,2]. EPS composition may differ due to bacterial strains, culture conditions, and biofilm age. Biofilm formation ensures antibiotic tolerance and protection from the host immune system, making microbial biofilms difficult to eradicate. Biofilms are responsible for chronic infections, and biofilms developed by a wide range of microorganisms are considered a “virulence factor” [3]. The variability in the composition of the biofilm matrix and in biofilm development, as well as tolerance versus the antimicrobials used in conventional therapies, suggest the need for multitargeted or combinational therapies aimed at the eradication of biofilms. Antimicrobial tolerance is due to different mechanisms such as the presence of an extracellular matrix that does not allow or slow the penetration of drugs as well as the presence of a metabolic dormancy adopted by many cells inside a biofilm. Furthermore, polymicrobial biofilms represent an additional problem that necessitates the use of antimicrobials that are efficacious versus all pathogens in biofilms, restricting the success of species-specific biofilm-targeting strategies [4,5].

The antibiofilm strategies proposed in the present special issue range from the use of antibiofilm peptides, the synthesis of derivatives starting from the structure of natural compounds, anti-quorum-sensing molecules, and nanosystems delivering inhibitors.

Among the molecules investigated with an antibiofilm effect, Dapunt et al. demonstrated that avian IgY could be capable of inhibiting quorum-sensing molecules (QSM) such as *Staphylococcus epidermidis* AtlE; a member of the autolysin family involved in the adhesion to a surface GroEL; a heat shock protein; and PIA, a polysaccharide intracellular adhesion responsible for cell–cell adhesion in biofilms. Scientists have demonstrated that IgY reduces biofilm formation, although the mechanism is not yet clear [6].

The antibiofilm activity of two antimicrobial peptides (AMPs), melamine and Mel4, and ciprofloxacin was tested against the *Pseudomonas aeruginosa* biofilm by Yasir et al. The results demonstrated that the minimum inhibitory concentration (MIC) of ciprofloxacin was not effective in inhibiting biofilm formation; on the contrary, AMPs reduced biofilm by 75% at their MIC values, depolarizing the cell membranes of biofilm cells. The association of AMPs with ciprofloxacin induced the release of a great amount of ATP but not of nucleic acids, suggesting that AMPs do not induce resistance in *P. aeruginosa* [7].

Of particular interest is the study of the antimicrobial and antibiofilm activities of both natural and synthetic compounds. In particular, Sisto et al. evaluated the activity of compounds based on the structure of thymol versus *Helicobacter pylori*. Three derivatives, also tested in order to verify a cytotoxic effect against AGS, showed antimicrobial activity and promising cytotoxicity, potentially representing new lead compounds alternative to current anti-*H. pylori* therapy aimed to prevent the occurrence of severe gastric diseases and gastric cancer. The present work confirms the possibility of using natural compounds as templates for medicinal semisynthesis [8].

Teichoic acids might represent another pharmacological key target. Teichoic acids are involved in important mechanisms such as Gram-positive growth, replication, and biofilm formation. Therefore, their inhibition represents a promising approach to eradicating biofilm-producing microorganisms. Naclerio et al. showed the capability of inhibitors of lipoteichoic acids (LTA) to halt the biofilm developed by methicillin-resistant *Staphylococcus aureus* and vancomycin-resistant *Enterococcus faecalis* [9].

Inhibition of the quorum-sensing system represents an additional appealing pharmacological target. Hallan et al. used liposomes to target two biofilm synthetic inhibitors. Different liposome formulations have been physicochemically characterized, and one of them was selected for the loading of biofilm inhibitors, demonstrating that liposomes can be used as a new strategy to increase the effect of inhibitors [10].

Bertoglio et al. tested the antimicrobial and antibiofilm activities of silver nanoparticles using silver fluoride as a precursor (AgF). The authors described both increased antibacterial and antibiofilm activities versus *Escherichia coli* and *Staphylococcus epidermidis* [11].

Regarding the use of natural compounds, Mohammed at al. characterized *Zygophyllum coccineum* phytoconstituents and subsequently tested the antimicrobial, antibiofilm, and anticancer bioactivities of both the plant extract and its fractions. Thirty-eight secondary metabolites belonging to flavonoid, stilbene, phenolic acid, alkaloid, and coumarin classes were identified as the main constituents. An in silico analysis of these constituents and their binding with Staph GyraseB and human topoisomerase-IIβ was performed to corroborate both the antimicrobial and anticancer biological activities [12].

The antifungal activity of the cinnamaldehyde has been demonstrated in in vitro and in situ assays on *Candida* spp. by da Nobrega Alves et al. [13]. The scientists showed both the antibiofilm activity and an effect on fungal morphology of cinnamaldehyde. Another strategy to fight *Candida* spp. infections and, in particular, the oral candidiasis is the use of ellagic acid-cyclodextrin complexes (EA/HP β-CD), as reported by da Graca Sampaio et al. [14]. The authors showed that EA/HP β-CD possess antifungal and anti-inflammatory activities suggesting its use as a promising treatment for oral candidiasis. Fungal dextranase obtained from *Penicillium roqueforti* and its encapsulation using alginate displayed a good antimicrobial activity versus *Streptococcus mutans*, suggesting the potential use of such formulation as an additive in toothpaste products to prevent dental caries [15].

Finally, the electrochemical activity of *Corynebacterium matruchotii*, a pathogen that is a component of oral biofilms, might be used for evaluating the effect of antimicrobial compounds. In their study, Naradasu et al. demonstrated that the efficacy of antimicrobials is evidenced by the suppression of microbial metabolic activity correlated with a significant current decrease. The method described may be applicable to the polymicrobial oral biofilms on the electrode surface [16].

In conclusion, advances in the micro- and nanotechnology fields have provided the production of innovative biomaterials and new strategies based on the use of new molecules/compounds used alone or in combination with antimicrobial delivery systems to fight biofilm development and/or eradicate biofilm-associated infections [17]. The use of new technologies in the clinical field is of great interest, as reported by De Meo et al., who provided a summary on the use of antibiotic-coated implants to reduce the risk of infection in orthopedic surgery [18].

## References

[1] Grande R., Nistico L., Sambanthamoorthy K., Longwell M., Iannitelli A., Cellini L., Stefano A.D., Stoodley L.H., Stoodley P. (2014). Temporal expression of *agrB, cidA*, and *alsS* in the early development of *Staphylococcus aureus* UAMS-1 biofilm formation and structural role of extracellular DNA and carbohydrates. Pathog. Dis..

[2] Grande R., Di Marcantonio M.C., Robuffo I., Pompilio A., Celia C., Di Marzio L., Hall-Stoodley L. (2015). Helicobacter pylori ATCC 43629/NCTC 11639 Outer Membrane Vesicles (OMVs) from biofilm and planktonic phase associated with extracellular DNA (eDNA). Front. Microbiol..

[3] Koo H., Allan R.N., Howlin R.P., Stoodley P., Hall-Stoodley L. (2017). Targeting microbial biofilms: Current and prospective therapeutic strategies. Nat. Rev. Microbiol..

[4] Carradori S., Di Giacomo N., Lobefalo M., Luisi G., Campestre C., Sisto F. (2020). Biofilm and Quorum Sensing inhibitors: The road so far. Expert Opin. Ther. Pat..

[5] Grande R., Puca V., Muraro R. (2020). Antibiotic resistance and bacterial biofilm. Expert Opin. Ther. Pat..

[6] Dapunt U., Prior B., Oelkrug C., Kretzer J.P. (2020). IgY Targeting Bacterial Quorum-Sensing Molecules in Implant-Associated Infections. Molecules.

[7] Yasir M., Dutta D., Willcox M.D. (2020). Activity of Antimicrobial Peptides and Ciprofloxacin against *Pseudomonas aeruginosa* Biofilms. Molecules.

[8] Sisto F., Carradori S., Guglielmi P., Spano M., Secci D., Granese A., Sobolev A., Grande R., Campestre C., Di Marcantonio M. (2021). Synthesis and Evaluation of Thymol-Based Synthetic Derivatives as Dual-Action Inhibitors against Different Strains of *H. pylori* and AGS Cell Line. Molecules.

[9] Naclerio G.A., Onyedibe K.I., Sintim H.O. (2020). Lipoteichoic Acid Biosynthesis Inhibitors as Potent Inhibitors of *S. aureus* and *E. faecalis* Growth and Biofilm Formation. Molecules.

[10] Hallan S.S., Marchetti P., Bortolotti D., Sguizzato M., Esposito E., Mariani P., Trapella C., Rizzo R., Cortesi R. (2020). Design of Nanosystems for the Delivery of Quorum Sensing Inhibitors: A Preliminary Study. Molecules.

[11] Bertoglio F., De Vita L., D’Agostino A., Fernandez Y.D., Falqui A., Casu A., Merli D., Milanese C., Rossi S., Taglietti A. (2020). Increased Antibacterial and Antibiofilm Properties of Silver Nanoparticles Using Silver Fluoride as Precursor. Molecules.

[12] Mohammed H., Khan R., Abdel-Hafez A., Abdel-Aziz M., Ahmed E., Enany S., Mahgoub S., Al-Rugaie O., Alsharidah M., Aly M. (2021). Phytochemical Profiling, In Vitro and In Silico Anti-Microbial and Anti-Cancer Activity Evaluations and Staph GyraseB and h-TOP-IIβ Receptor-Docking Studies of Major Constituents of *Zygophyllum coccineum* L. Aqueous-Ethanolic Extract and Its Subsequent Fractions: An Approach to Validate Traditional Phytomedicinal Knowledge. Molecules.

[13] Alves D.D.N., Monteiro A.F.M., Andrade P.N., Lazarini J.G., Abílio G.M.F., Guerra F.Q.S., Scotti M.T., Scotti L., Rosalen P.L., De Castro R.D. (2020). Docking Prediction, Antifungal Activity, Anti-Biofilm Effects on *Candida* spp., and Toxicity against Human Cells of Cinnamaldehyde. Molecules.

[14] Sampaio A., Gontijo A., Lima G., de Oliveira M., Lepesqueur L., Koga-Ito C. (2021). Ellagic Acid–Cyclodextrin Complexes for the Treatment of Oral Candidiasis. Molecules.

[15] Juntarachot N., Kantachote D., Peerajan S., Sirilun S., Chaiyasut C. (2020). Optimization of Fungal Dextranase Production and Its Antibiofilm Activity, Encapsulation and Stability in Toothpaste. Molecules.

[16] Naradasu D., Miran W., Okamoto A. (2020). Metabolic Current Production by an Oral Biofilm Pathogen *Corynebacterium matruchotii*. Molecules.

[17] Rao H., Choo S., Mahalingam S.R., Adisuri D., Madhavan P., Akim A.M., Chong P. (2021). Approaches for Mitigating Microbial Biofilm-Related Drug Resistance: A Focus on Micro- and Nanotechnologies. Molecules.

[18] De Meo D., Cannari F.M., Petriello L., Persiani P., Villani C. (2020). Gentamicin-Coated Tibia Nail in Fractures and Nonunion to Reduce Fracture-Related Infections: A Systematic Review. Molecules.

